# Study on the Properties of All-Solid Waste Fluidized Filling Materials Applied to Mine Void Area Filling Engineering

**DOI:** 10.3390/ma17215154

**Published:** 2024-10-23

**Authors:** Yuting Lu, Junjie Yang, Yalei Wu, Ruifan Lu, Yunhong Li, Lixiang Zhang, Jiangtao Guo

**Affiliations:** 1 College of Environmental Science and Engineering, Ocean University of China, Qingdao 266100, China; luyuting@stu.ouc.edu.cn (Y.L.); jjyang@ouc.edu.cn (J.Y.); lruifan00@163.com (R.L.); 2 The Key Laboratory of Marine Environment and Ecology of the Ministry of Education, Ocean University of China, Qingdao 266100, China; 3 Shandong Bureau Group Qingdao Co., Ltd. of China Metallurgical Geology Bureau, Qingdao 266109, China; wfdsxmb@126.com (Y.L.); 18661774305@163.com (L.Z.); 4 Laizhou Jiujiang Construction Engineering Co., Yantai 261400, China; 13287951158@163.com

**Keywords:** flowable filling material, solid waste, fluidity, constructability, strength

## Abstract

The extraction of mining resources, as well as processing processes such as ore beneficiation and smelting, generate large amounts of tailings that are difficult to directly utilize. Meanwhile, substantial filling materials are required for the voids formed after mining operations, and the environmental issues and safety hazards brought on by massive solid waste disposal cannot be ignored. By utilizing solid waste with alkaline and pozzolanic activity as the binder component and gold tailings as filler aggregate to prepare filler material to fill up the void areas, the purpose of waste treatment can be achieved. In this study, salt sludge, steel slag, ground granulated blast furnace slag, and gold tailings were used to prepare all-solid waste fluidized filling material for filling mine void areas, which not only solves the engineering safety problem of easy collapse of the mine airspace in the mining process but also ensures a backfill effect with high strength, which continuously guarantees the uninterrupted progress of the mining project. At the same time, the preparation of fluidized materials can consume a large amount of tailings and other solid waste, solving the problem of their stockpiling. The components of the solid wastes used are all general industrial solid wastes, so the preparation of the fluidized materials will not have an impact on the surrounding environment. The effects of binder ratios on the workability of the filling materials were investigated by means of the slump and slump flow tests. Combined with the unconfined compressive strength test, the change in backfill material strength with curing age and the water–binder ratio was studied. The experimental results showed that the slump and slump flow value of the filling material were positively correlated with the water–binder ratio. The water–binder ratio range satisfying a slump value of 180~260 mm and a slump flow value not less than 400 mm was 0.95~1.106. However, the strength decreased with the increase in the water–binder ratio, conforming to a hyperbolic relationship. The all-solid waste fluidized filling material had strengths not less than 0.22, 1.09, and 1.95 MPa at 3, 7, and 28 d, respectively, meeting the workability requirements. Finally, a method for determining the optimal range of water–binder ratio considering both workability performance and strength is proposed based on the relationship between slump value, slump flow value, unconfined compressive strength, and the water–binder ratio.

## 1. Introduction

With the rapid growth of China’s economy, the demand for natural resources is expanding, which greatly promotes the rapid development of mining enterprises. The continuous development of mining resources has produced a large number of hollow areas, and the subsidence instability of hollow areas is a problem that needs to be solved urgently. Filling technology is an effective technology to transport filling materials underground to fill the hollow area under the action of an external force or self-weight. Filling materials are generally made of binder and filling aggregate mixed with water. Cement is widely used as a traditional binder; however, its production process brings about energy consumption, resource consumption, ecological damage, and environmental pollution problems that cannot be ignored [1,2]. On the other hand, tailings from mining resource extraction and processing, such as ore dressing and smelting [3], salt sludge from the chlor-alkali industry [4], steel slag and ground granulated blast furnace slag from the iron and steel industry [5,6], and other industrial solid wastes are increasing day by day, which is not only a serious land use problem but also a hazard to the natural environment.

The main components of salt sludge are calcium carbonate, magnesium hydroxide, and sodium chloride, among which calcium carbonate has the highest content. Calcium carbonate has strong hygroscopicity and can react with water to generate calcium bicarbonate, which is further converted to calcium hydroxide, so it has the potential to be used as an alkaline activator [7]. However, there has not yet been any research on it as a component of the cementitious material. GGBS is a good alkaline-activated material, which has been intensively studied and is widely used in engineering [8,9]. The main components of steel slag include C3S, C2S, C4AF, and C2F, which can form calcium silicate hydrate (CSH) when reacting with water, showing good cementing activity [10]. SS can also replace some highly active cementing materials when stimulated by alkaline solutions, so the use of steel slag as a supplementary cementing material is also one of the main utilization routes [11,12]. The use of the above solid wastes to prepare filling materials to fill up the mining area has significant economic and environmental benefits.

Generally, filling is the pumping of filling material to the voided area by gravity or pipeline, so it is necessary that the filling material has workability (slump and slump flow). At the same time, the backfill formed by the maintenance of the filling material should have a certain degree of strength [13,14]. Du et al. [15] used slag, lime, gypsum, cement, and a small amount of admixtures as components to prepare a fluidized high-strength filling material for total tailings, which can reduce the cost of total tailing filling mining. Chen et al. [16] used cement/fly ash/tailings at a ratio of 1:2:8 to prepare a filling body with a strength of up to more than 2 MPa at 90 d. Dong et al. [17] studied the effect of fly ash dosing on the performance of filling materials using fly ash, ground granulated blast furnace slag, lime, and desulfurization gypsum as the binder components and found that high-concentration self-flow transport of filling materials could be achieved when 10–20% fly ash was dosed and the 28 d strength of the backfill could meet the requirements of filling mining. Jiang et al. [18] investigated the effects of three different admixtures, namely fly ash (FA), granulated blast furnace slag (GGBS), and silica fume (SF), on the thixotropy and rheology of the filling materials and the strength of the filling body, respectively, using cement as the binder and tailings as the filling aggregate. Zhao et al. [19] used low-calcium fly ash (FA), sodium silicate solution, and soda residue (SR) to prepare fluidized filling materials for the hollow zone and obtained a filling material with a slump of 260 mm and a 28 d strength of the backfill of 3.70 MPa when FA/SR = 3:2 and the concentration of sodium silicate solution was 2.0 mol/L. Zeng et al. [20] used ordinary silicate cement, shield sludge, foam, and activated magnesium oxide to prepare filling materials. By adjusting the dosage of foam agent and active magnesium oxide, a filling material with a flow degree of 180~320 mm, a water secretion rate of less than 5%, and a 28 d strength of 0.6~1.2 MPa could be obtained. Yin et al. [21] used raw phosphor gypsum (PG) as filler, and yellow phosphorus slag (YPS), fly ash (FA), steel slag (SS), and Portland cement were mixed to make a composite binder. By analyzing the effects of adding different proportions of solid waste and curing time on the flow properties and strength of the backfill, it was verified that it was feasible to use solid waste for mine backfill. Xie et al. [22] used gangue, cement, fly ash, and water to make filling materials and proposed the process flow of filling technology in the hollow zone and the selection method of the technology model.

The use of multiple solid wastes in the production of cementitious materials has been widely studied [23]. The use of slag and steel slag as components of cementitious materials has been widely studied. Wu et al. [24] studied the preparation of electrolytic manganese slag-based cementitious materials (ESGM) using electrolytic manganese slag as a raw material combined with steel slag (SS) and blast furnace slag (GGBS) in the presence of an alkali activator (NaOH). The compressive strength reached its maximum value of 23.0 MPa at 28 d when the ratio of EMR/SS/GGBS was 5:2:3 and the concentration of the alkali activator was 0.2. Lin et al. [25] used steel slag (SS), ground granulated blast furnace slag (GGBS), and fly ash (FA) to synergistically produce alkali-activated composite materials (AAMs). The effects of different ratios of the components on the strength and flow of the materials were discussed. The best tensile deformation properties were achieved at about 15% SS. Wu et al. [26] developed an all-waste CGF binder using calcium carbide slag as an alkali exciter and blast furnace slag and fly ash as pozzolanic materials. They revealed the mechanical properties and curing mechanism of pure clay, pure silt, and pure sand cured by CGF through macroscopic and microscopic experiments and quantitatively assessed the environmental and economic benefits of an all-waste CGF binder. Yan et al. [27] used CGF binder to prepare all-solid waste fluid-cured soil for offshore wind power pile foundation scour protection.

Mine filling mostly uses pumping or pipelines to pour the fluidized filling materials into the mine void areas. In order to ensure the strength of the backfilled area and prevent the mining project from being affected by the backfilling of the area, the workability and strength properties are the most important aspects of mine filling materials. It is not yet known whether the performance of all-solid waste fluidized filling material can meet the engineering requirements. In view of this, this study utilized salt sludge (SAS), steel slag (SS), ground granulated blast furnace slag (GGBS), and gold tailings to prepare all-solid waste fluidized filling material for mine void area filling. By conducting slump and slump flow tests, the influence of the water–binder ratio on the workability of the filling material was investigated. Combined with unconfined compressive strength tests, the variation in the strength of the backfill with curing age and the water–binder ratio was studied. Based on these findings, a method for determining the optimal range of water–binder ratio that balances workability and strength is proposed, providing a scientific basis for the application of all-solid waste fluidized filling material in mine void area filling.

## 2. Materials and Methods

### 2.1. Materials

**Figure 1:**
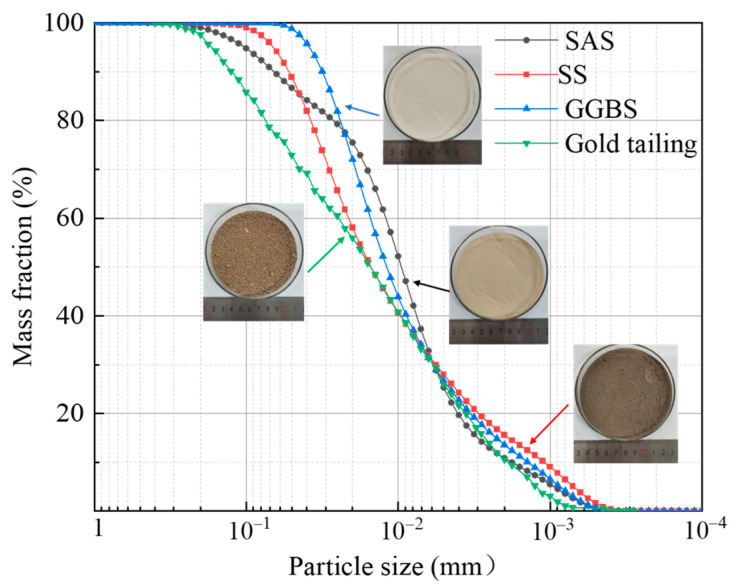
Particle size distribution curves of the testing materials.

**Figure 2:**
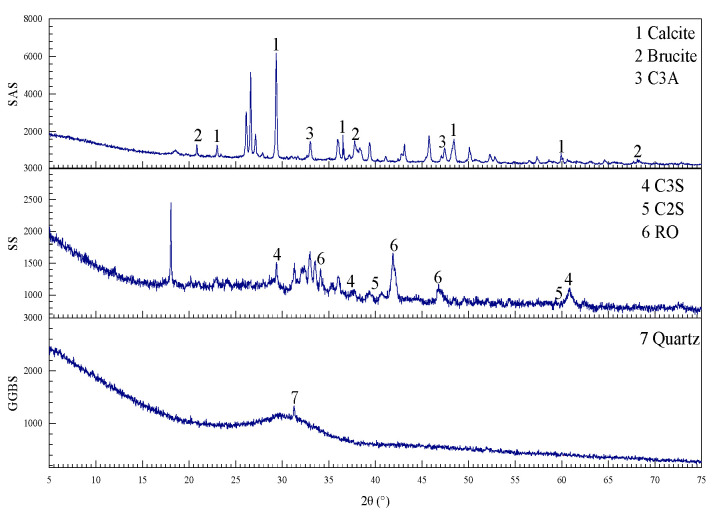
XRD patterns.

The testing materials included salt sludge (SAS), steel slag (SS), ground granulated blast furnace slag (GGBS), and gold tailings. Among these, SAS, SS, and GGBS served as the binder components, while gold tailings served as the filling aggregates. The binder was mixed with the filling aggregates and water to form the all-solid waste fluidized filling material. The SAS was sourced from a chemical plant in Weifang, Shandong Province (Weifang, Shandong, China). The SS, GGBS and gold tailings were taken from the steel mill and gold tailings dumps (Yantai, Shandong, China), respectively. The grading curves of the four raw materials are shown in Figure 1, and the curing agent components (salt industrial waste, steel slag, and slag) measured by XRD are shown in Figure 2.

The specific gravity of the gold tailings was 2.69. The maximum particle diameter of the gold tailings was 1 mm, with a particle content smaller than 0.075 mm of 7.6%, categorizing it as containing fine-grained silt [28]. The moisture content of the testing materials is shown in Table 1. The cumulative particle size distribution curve is depicted in Figure 1. A coefficient of uniformity (Cu) of 13.36 and a coefficient of curvature (Cc) of 0.76 indicated poor-grade sand.

The chemical composition of the binder components (SAS, SS, and GGBS) obtained by XRF analysis is shown in Table 2.

The main components of salt sludge are magnesium hydroxide, calcium carbonate, and a small amount of brine. Additionally, the salt sludge used in this experiment had been treated to reduce the chloride ion content to less than 1%.

### 2.2. Schemes

The binder consisted of salt sludge, steel slag, and ground granulated blast furnace slag as components, with a mass ratio of 2:5.5:2.5. The mass ratio of the binder to gold tailings was 2:10, corresponding to a blending ratio of 20%. The all-solid waste fluidized bed filling material was formed by mixing the binder with gold tailings and water. The filling material was sampled and cured to a set curing age to form the backfill, and the test scheme is shown in Table 3.

### 2.3. Methods

According to the provisions of ‘Apparatus for Concrete Slump Test’ [29], a slump cone with an upper inner diameter of 100 mm, lower inner diameter of 200 mm, and height of 300 mm was used for the workability test. Following the experimental plan outlined in Table 3, the solid waste was weighed and mixed with water to obtain the all-solid waste fluidized filling material. Subsequently, the workability test was conducted to measure the slump and slump flow value of the material. After completing the workability test, cylindrical specimens for unconfined compressive strength testing were prepared with dimensions of 100 mm height and 50 mm diameter. Three sets of parallel specimens were prepared. The specimens were demolded after standard curing for 1 d (curing temperature: 20 ± 2 °C, relative humidity ≥ 98%) and then wrapped in plastic film and placed in a curing chamber for continued standard curing until the designated curing ages (3, 7, and 28 d). The test equipment used a WCY-1-type stress–strain-controlled unconfined pressure gauge produced by Nanjing Zhongzhiyan Measurement and Control Technology Co., Ltd. (Nanjing, China) to carry out unconfined compressive strength tests, and unconfined compressive strength tests were conducted at a loading rate of 1 mm/min.

## 3. Results and Discussion

### 3.1. Filling Material Workability

**Figure 3:**
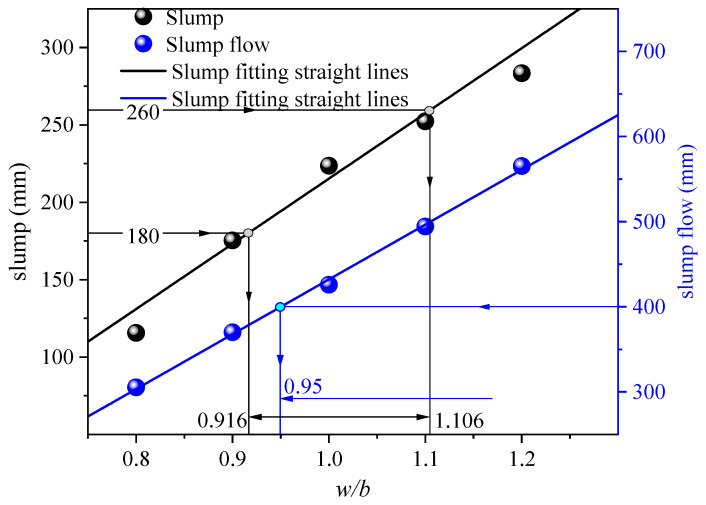
Effect of the water–binder ratio on the workability of filling material.

The variation trend of the workability of all-solid waste fluidized filling material with the water–binder ratio is shown in Figure 3. Within the range of water–binder ratios considered in this study, both slump value and slump flow value increased with the increase in the water–binder ratio, showing an approximately linear trend. This result is consistent with the experimental findings of other researchers [30,31].

In this study, the relationship between slump and slump flow value with the water–binder ratio was fitted linearly separately, yielding the following equations:

(1) $$\begin{array}{l} \mathrm{sl}=421.4w/b-206.08\\ \mathrm{fl}=644.1w/b-211.94 \end{array}$$

where *sl* represents the slump value in millimeters, *w*/*b* denotes the water–binder ratio, and *fl* signifies the slump value in millimeters.

To meet the workability requirements of fluidized filling material, it is generally recommended that the slump falls within the range of 180~260 mm and the slump flow value is not less than 400 mm [32]. As shown in Figure 3, the water–binder ratios that met the slump requirement ranged from 0.916 to 1.106, while the water–binder ratio that satisfied the slump flow requirement was greater than 0.95. Therefore, under the experimental conditions of this study, the water–binder ratio that met the workability criteria was 0.95~1.106.

### 3.2. Unconfined Compressive Strength of Backfill

**Figure 4:**
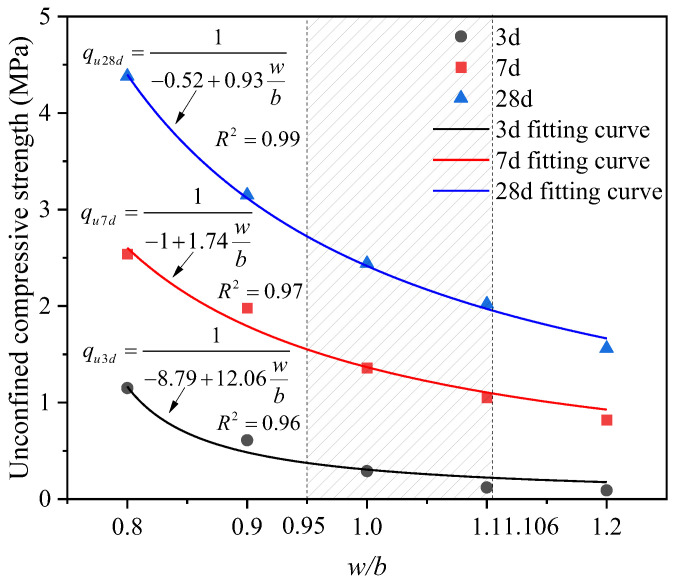
Effect of water–binder ratio on the unconfined compressive strength of backfill.

The variation trend of unconfined compressive strength of backfill with the water–binder ratio is shown in Figure 4. As the water–binder ratio increased, the unconfined compressive strength gradually decreased. When the water–binder ratio increased from 0.8 to 1.2, the strength decreased by 47.0%, 74.8%, 89.6%, and 92.2%, respectively, at curing of 3 d. Similarly, the strength decreased by 22%, 46.5%, 58.7%, and 67.7% at curing of 7 d, while the strength decreased by 28.1%, 44.3%, 53.9%, and 64.4% at curing of 28 d. The higher the water–binder ratio, the more water exceeds the amount required for the hydration reaction, resulting in lower strength accordingly [33,34].

Therefore, the water–binder ratio is a crucial factor affecting the strength of fluidized filling material. The relationship between unconfined compressive strength and water–binder ratio is fitted with a hyperbolic curve, yielding a functional relationship between the water–binder ratio and compressive strength, as shown in Equation (2).

(2) $$q_{u}=\frac{1}{m+n(w/b)}$$

where *q_u_* represents the unconfined compressive strength, and *m* and *n* are the fitting parameters. The *w*/*b* is the same as in Equation (1).

After fitting the strength data for curing ages of 3, 7, and 28 d using Equation (2), the corresponding coefficient of determination (R^2^) values were 0.956, 0.973, and 0.997, respectively. This indicates that the relationship between unconfined compressive strength and water–binder ratio conformed to a hyperbolic curve.

According to Figure 4, under the experimental conditions of this study, the minimum strengths at 3, 7, and 28 d corresponding to the water–binder ratios of 0.95–1.106, which satisfy the workability requirements, reached 0.22, 1.09, and 1.95 MPa, respectively.

**Figure 5:**
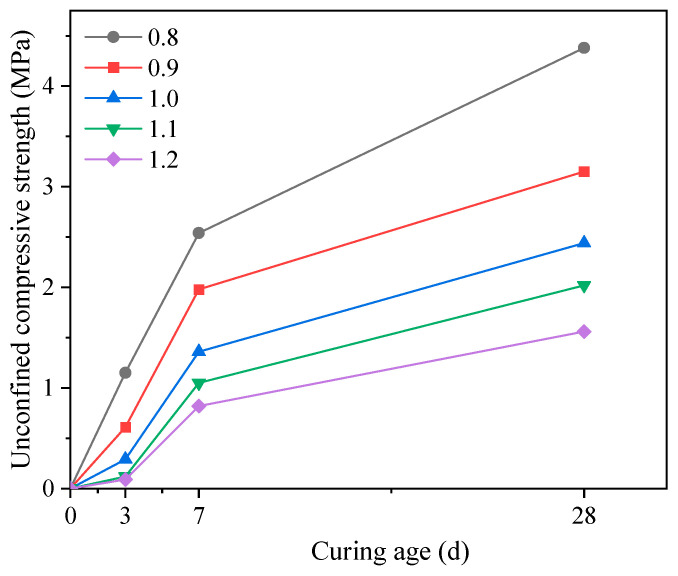
Effect of curing age on the unconfined compressive strength of backfill.

The influence of curing age on the unconfined compressive strength of backfill is depicted in Figure 5. The strength increased with the curing age, with a noticeable increase from 0 to 3 d, a significant increase from 3 to 7 d, and a slowing down of strength growth from 7 to 28 d. Moreover, this growth pattern was not influenced by the water–binder ratio. As the curing age progressed, the reaction between the components of the binder gradually intensified. After 7 d, the strength development was mainly attributed to the continuous hydration reaction. However, after a certain curing period, the hydration reaction slowed down, leading to a corresponding slowdown in strength growth [21,35].

### 3.3. Discussion

In this study, all-solid waste fluidized filling materials were prepared using SAS, SS, GGBS, and gold tailings, and the workability and strength properties of the fluidized materials were investigated by slump and slump flow tests as well as unconfined compressive strength tests. It was concluded that the water–binder ratio showed a positive correlation with the slump and slump flow value, while the strength showed a hyperbolic relationship with the increase in the water–binder ratio. The effect of age on strength was not affected by the water–binder ratio.

Based on the existing studies, it has been found that the key to realizing large-scale underground backfilling with solid waste slurry is the workability and early strength of the backfill material [36,37]. The flowability affects the delivery of the filling slurry and the backfilling efficiency, while the early strength affects the filling of the voided area after backfilling. 

Fall et al. [38] and others proposed a mixed-design method for cemented tailing fillers by taking the water–binder ratio of cemented tailing fillers as well as compressive strength and slump value as the indexes, using ANOVA to show that the water–binder ratio had a significant effect on slump as well as unmeasured compressive strength. Gao et al. [39] prepared a green thixotropic cement paste backfill with molybdenum tailing (MOT) sand and proposed a thixotropic coefficient method to evaluate the rheology as well as the compressive strength of the paste. The analysis showed that the viscosity of the paste decreased with the increase in the water-cement ratio, implying an increase in fluidity, and the mechanical properties of the stone body decreased gradually with the increase in the water–binder ratio. Finally, the optimum water–binder ratio was obtained as 0.8. This is consistent with the results of our study.

However, in previous studies, more attention has been paid to how solid waste components, water–binder ratio, etc., affect the performance of the material. This paper further proposes a determination method to determine the range of water–binder ratios that meet the requirements according to the needs of actual projects, which puts the theory into practice and provides certain guidance for the project.

**Figure 6:**
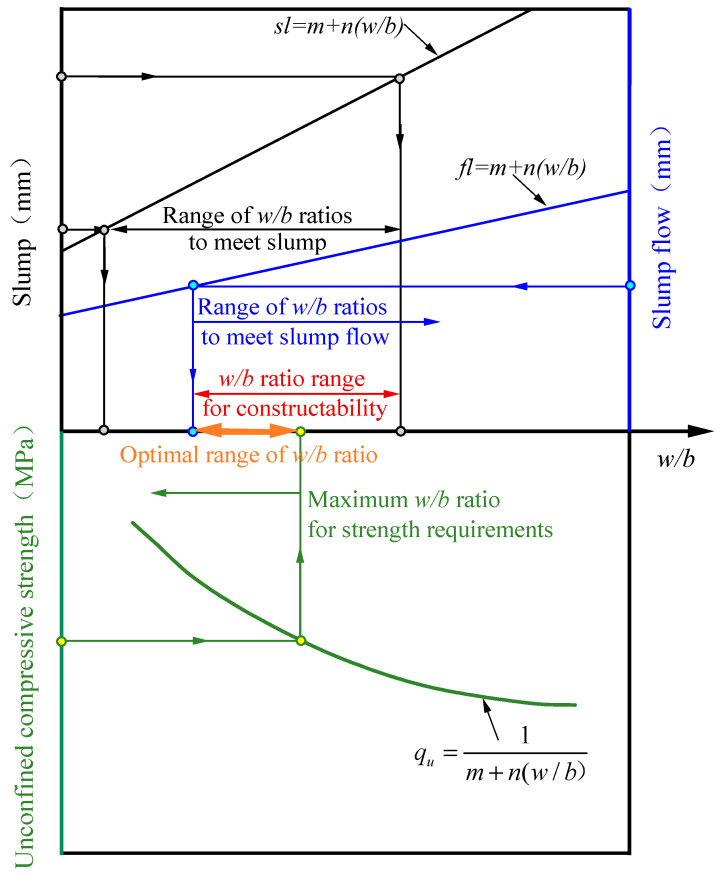
Method for determining the optimal range of the water–binder ratio.

The following method is proposed to determine the optimal range of water-binder ratio that balances workability and strength, as illustrated in Figure 6. As the filling material should meet the workability requirements, firstly, the slump and slump flow test of the filling material must be carried out to obtain the slump and slump flow values under different water–binder ratio conditions [33,34]. Curve fitting is then carried out for the data under each test condition, and the range of water-binder ratios to meet the workability requirements is determined according to the specifications of the slump and slump flow values and the engineering requirements.

Secondly, the backfill formed after maintenance of the filling material should meet the strength requirements. The unconfined compressive strength of the fluidized filling material is controlled to a certain extent by the water–binder ratio, and when the amount of water meets the required range of the activation reaction of the curing agent, a smaller water–binder ratio is needed to obtain a higher unconfined compressive strength [21,35]. After fitting the relationship between the unconfined compressive strength of the backfill and the water–binder ratio, the maximum water–binder ratio is obtained, corresponding to the minimum strength required by the project.

The larger the water–binder ratio, the greater the slump and slump-flow values but the lower the strength [39,40,41]. The optimum range of water–binder ratios to meet engineering requirements needs to be a range that meets both workability and strength requirements.

The limitation of this study is that it determines the range of water-binder ratios only through macroscopic experimental analyses and analyses of practical engineering applications, without in-depth chemical analyses in terms of microscopic mechanisms. Moreover, the specific influence of SAS in the hydration reaction of the curing agent has not yet been clarified. Secondly, the durability performance of this all-solid waste fluidized material needs to be further investigated.

## 4. Conclusions

This study utilized salt sludge, steel slag, ground granulated blast furnace slag, and gold tailings to prepare filling material for mine void areas and investigated their filling performance through slump, slump flow, and unconfined compressive strength tests. The main conclusions are as follows:

(1) Under the water–binder ratio conditions in this study, the slump and slump flow value of all-solid waste fluidized filling material were positively correlated with the water–binder ratio. When the water–binder ratio was in the range of 0.95 to 1.106, the filling material met the workability requirements of slump value between 180 and 260 mm and slump flow value not less than 400 mm.
(2) The unconfined compressive strength of the backfill decreased with increasing water–binder ratio, conforming to a hyperbolic relationship. The strength gradually increased with the curing age, with noticeable increases from 0 to 3 d, significant increases from 3 to 7 d, and a slowing down of strength growth from 7 to 28 d. This growth pattern was not influenced by the water–binder ratio.
(3) Under the experimental conditions of this study, the 3, 7, and 28 d backfill strengths of the all-solid waste fluidized filling materials that satisfy workability requirements were not less than 0.22, 1.09, and 1.95 MPa, respectively.
(4) Based on the relationship between slump value, slump flow value, unconfined compressive strength, and water–binder ratio, a method for determining the optimal range of water–binder ratio that balances workability and strength is proposed.

## Figures and Tables

**Table 1:** Initial moisture content of the testing materials (%).

SAS	SS	GGBS	Gold Tailing
56.9	0.2	0.3	0.5

**Table 2:** Chemical composition of the testing materials.

All-Solid Waste Fluidized Filling Material		Chemical Composition and Content (%)								
		SiO_2_	CaO	Al_2_O_3_	SO_3_	Fe_2_O_3_	MgO	K_2_O	Na_2_O	LOI
Binder	SAS	1.18	30.96	0.31	0.81	0.85	24.16	8.64	0.59	32.5
	SS	14.84	48.64	3.72	0.64	27.55	1.21	0.56	0.04	2.8
	GGBS	30.87	41.86	14.97	2.50	0.34	8.05	0.27	0.32	0.82
Filler aggregate	Gold tailing	74.8	0.89	9.91	0.4	3.62	0.17	9.11	0.17	0.93

**Table 3:** Test schemes and backfill tests.

Water–Binder Ratio	Curing Age (d)	Tests
0.8	3, 7, 28	Slump, slump flow, Unconfined compressive strength
0.9		
1.0		
1.1		
1.2		

## Data Availability

The data presented in this study are available upon request from the corresponding author. The data are not publicly available due to the confidentiality of the research subject.
